# ﻿A new classification of C4- *Atriplex* species in Russia, with the first alien record of *Atriplexflabellum* (Chenopodiaceae, Amaranthaceae) from North Siberia

**DOI:** 10.3897/phytokeys.202.87306

**Published:** 2022-07-22

**Authors:** Alexander P. Sukhorukov, Maria Kushunina, Alexander N. Sennikov

**Affiliations:** 1 Department Higher Plants, Biological Faculty, Moscow State University, Leninskie Gory 1/12, Moscow, 119234, Russia; 2 Laboratory Herbarium (TK), Tomsk State University, Lenin Ave. 36, Tomsk, 634050, Russia; 3 Department Plant Physiology, Biological Faculty, Moscow State University, Leninskie Gory 1/12, Moscow, 119234, Russia; 4 Botanical Museum, Finnish Museum of Natural History, P. O. Box 7, 00014 University of Helsinki, Helsinki, Finland

**Keywords:** Alien species, *Atriplexflabellum*, distribution, Russia, systematics, taxonomy

## Abstract

For a long time, the systematics of *Atriplex* was based solely on morphological characters and leaf anatomy. The latest worldwide phylogenetic study of *Atriplex* significantly improved our knowledge about the relationships within the genus, but a new classification has not been put forward thus far. Here we re-evaluate the taxonomy of C_4_-species of *Atriplex* that are native to Russia. Seven species are classified into two sections, A.sect.Obione (incl. A.sect.Sclerocalymma, syn. nov.) (*A.altaica*, *A.centralasiatica*, *A.rosea*, *A.sibirica*, and *A.sphaeromorpha*), and A.sect.Obionopsis (incl. A.sect.Psammophila, syn. nov.) (*A.fominii* and *A.tatarica*). Although the majority of Eurasian C_4_-species have similar morphology, leafy inflorescence is a typical character for A.sect.Obione. The members of A.sect.Obionopsis are characterised mostly by aphyllous inflorescences, but some species (*A.laciniata*, *A.pratovii*, and *A.tornabenei*) have leafy inflorescences. Geographically, almost all members of A.sect.Obione are confined to Central Asia, although *A.rosea* is a typical Mediterranean element and *A.argentea* occurs in North America. The representatives of A.sect.Obionopsis are distributed mostly in the Mediterranean and the Irano-Turanian floristic region. The alien status of *A.rosea*, *A.sibirica* and *A.tatarica* is discussed. *Atriplexflabellum*, a desert species from the Irano-Turanian region, is reported for the first time from Russia (Yamalo-Nenets Autonomous District, North Siberia) as a casual alien. This species occupies a phylogenetic position distant from both aforementioned sections. An identification key to all C_4_-species of the genus growing in Russia is given, and a sectional checklist with updated nomenclature and revised synonymy is provided.

## ﻿Introduction

*Atriplex* L. is the largest genus in the subfamily Chenopodioideae (Amaranthaceae*sensu*APG IV 2016), after *Chenopodium* L. s.l. has been disassembled into several genera of different taxonomic placement within the subfamily (Fuentes-Bazan et al. 2012). It encompasses ca. 260 species distributed worldwide (Žerdoner Čalasan et al. 2022). In Eurasia, many species of *Atriplex* are found in deserts, especially in the Irano-Turanian floristic region, and it was suggested that the genus originated in continental Asia (Žerdoner Čalasan et al. 2022).

The classification system of *Atriplex* had been at first based on morphological characters (e.g., Aellen 1939; Wilson 1984); additionally, leaf anatomy was used as an important character for the delimitation of *Atriplex* species. The genus was divided into two physiological groups: with non-Kranz (C_3_) and Kranz (C_4_) anatomy (Moser 1934; Carolin et al. 1975; Welsh 2001; Sukhorukov 2006). In the latest treatment for Russia and adjacent countries (Sukhorukov 2006), *Atriplex* was divided into two subgenera based on the fusion of the perianth segments (valves) in the female flowers: A.subgen.Atriplex with marginally connate valves and A.subgen.Pterotheca (Aellen) Sukhor. with ventrally fused valves. The valves imitate bracteoles and pairs of them form bract-like covers enclosing each female flower. The type subgenus comprised several sections, characterised by the degree of valve fusion and their sclerification, leaf anatomy, the type of diurnal leaf movements, as well as their fruit and seed characters.

Molecular phylogenetic studies (Kadereit et al. 2010; Žerdoner Čalasan et al. 2022) confirmed a close relationship of many Eurasian C_3_-species, especially those of A.sect.Atriplex and A.sect.Teutliopsis Dumort. emend. Sukhor. (excl. *A.oblongifolia* Waldst. & Kit.), as well as a distant position of *A.cana* C.A.Mey. from other members proposed by Sukhorukov (2006). All C_4_-species of the genus comprise a monophyletic clade (Kadereit et al. 2010; Žerdoner Čalasan et al. 2022) with ca. 180 members distributed in the arid and mountainous regions around the world (Sage 2016).

In Eurasia, the majority of C_4_-species were traditionally united into A.sect.Sclerocalymma (Asch.) Asch. & Graebn. and A.sect.Obione (Gaertn.) Reichenb., differing mainly by the degree of valve fusion (Iljin 1936; Aellen 1939; Medvedeva 1996; Sukhorukov 2006). Atriplexsect.Sclerocalymma included annual species with the valves fused to (nearly) half of their length (*A.altaica* Sukhor., *A.fominii* Iljin, *A.kalafganica* Podlech, *A.laciniata* L., *A.megalotheca* Popov, *A.olivieri* Moq., *A.pallida* (Moq.) Sukhor. [= *A.schugnanica* Iljin], *A.pamirica* Iljin, *A.paradoxa* Nikitina, *A.pratovii* Sukhor., *A.pungens* Trautv., *A.recurva* D’Urv., *A.rosea* L., *A.sphaeromorpha* Iljin, *A.tatarica* L., *A.tianschanica* U.P.Pratov, and *A.tornabenei* Tineo). Atriplexsect.Obione comprised *A.belangeri* (Moq.) Boiss., *A.centralasiatica* Iljin and *A.sibirica* L. (Sukhorukov 2006, 2014).

Nearly all of the aforementioned species (except *A.belangeri* and *A.tianschanica*) were included in the latest molecular phylogenetic study of the genus, and they fell into two phylogenetic lineages with different positions on the tree within the large C_4_-group (Žerdoner Čalasan et al. 2022). The previous morphology-based classification (Sukhorukov 2006, 2014) only partly agrees with the phylogenetic relationships. Thus, the systematics of *Atriplex* needs to be revised, with the re-evaluation of its section-level taxonomy.

The present article is dedicated to a new sectional subdivision of the C_4_-species of *Atriplex* growing in Russia as the first step towards a new taxonomic classification of *Atriplex* worldwide. It summarises the distributional data for all its members including new, unexpected alien occurrences, with further notes on geographical patterns revealed in the new classification.

## ﻿Materials and methods

Our new classification of C_4_-*Atriplex* taxa occurring in Russia is based on the most recent phylogenetic study (Žerdoner Čalasan et al. 2022) and follows the principle of strict monophyly.

Historical taxonomic literature was examined for infrageneric classifications in *Atriplex*. A section-level taxonomic and nomenclatural checklist was compiled in order to evaluate the infrageneric names used to classify the species under study. Taxonomic literature was screened for protologues, which were evaluated according to the current rules of botanical nomenclature (Turland et al. 2018).

We used the distribution data for each species given by Hedge (1997) and Sukhorukov (2006, 2014), which were complemented by our recent field and herbarium studies. Herbarium collections from LE, LECB, MHA, MW, MSK, MSKU, MWG, NS and NSK were critically revised for taxonomic identifications and screened for new records.

Distribution maps were prepared using SimpleMappr online tool (http://www.simplemappr.net) based on the literature data and the examined specimens. The results were generalised and schematically presented over the basemap of first-level administrative subdivisions of Russia. Distribution areas were evaluated for their native core and secondary dispersal based on herbarium specimens and our personal observations in the field, thus separating the territories where the species occur in their natural habitats from those where the species are confined exclusively to ruderal or other man-made habitats.

## ﻿Results and discussion

### ﻿Taxonomy of the native species

The C_4_-species of *Atriplex* native to Russia are here classified into two sections, A.sect.Obione (Gaertn.) Reichenb. and A.sect.Obionopsis (Lange) Dumort., according to their phylogenetic position (Žerdoner Čalasan et al. 2022). The latter sectional name had been forgotten for a long time but is resurrected here as the earliest name applicable to the group that includes its type species *A.laciniata* and related taxa. In the checklist, previously overlooked protologues are cited for accepted names together with second references (other than protologues) that indicate the works previously considered as places of valid publication.

#### ﻿ Atriplex sect. Obione

Taxon classificationPlantaeCaryophyllalesChenopodiaceae

(Gaertn.) Reichenb., Uebers. Gew.-Reich.: 164 (1828); C.A.Mey. in Ledeb. et al., Fl. Altaic. 4: 315 (1833).

42F0D9AF-984D-50A0-86F0-F2D5670A7F05

≡ Obione Gaertn., De Fruct. 2: 198 (1791).
≡ Obionesect.Atriplicina Moq., Chenop. Monogr. Enum.: 70 (1840), nom. inval. (Art. 22.2).
≡ Atriplexsubgen.Obione (Gaertn.) Hook.f., Student Fl. Brit. Isl.: 320 (1870); Volkens in Engler & Harms, Nat. Pflanzenfam. 3: 66 (1893).
≡ Atriplexsect.Atriplicina Volkens in Engler & Harms, Nat. Pflanzenfam. 3: 66 (1893), nom. illeg. (Art. 52.1).
≡ Obionesect.Protobione Aellen, Verh. Naturf. Ges. Basel 49: 133 (1938), nom. inval. (Art. 22.2). Type species: Obionemuricata Gaertn. (≡ Atriplexsibirica L.).
= Atriplex [unranked] Sclerocalymma Asch., Fl. Prov. Brandenburg 1(2): 578 (1864), syn. nov.
≡ Atriplexsect.Sclerocalymma (Asch.) Asch. & Graebn., Syn. Mitteleur. Fl. 5(1): 139 (1919).
≡ Atriplexsect.Roseae Aellen, Bot. Jahrb. Syst. 70(1): 39 (1939), “Rosea”, nom. illeg. (Art. 52.1). Type species: Atriplexrosea L.
= Atriplex [unranked] Argenteae Standl. in Britton, N. Amer. Fl. 21: 46 (1916), syn. nov.
≡ Atriplexsubsect.Argenteae (Standl.) S.L.Welsh, Rhodora 102: 420 (2001). Type species (Art. 10.8): Atriplexargentea Nutt.

##### Description.

Annuals; inflorescences leafy; glomerules loosely arranged.

##### Native distribution and species.

**Figure 1.**
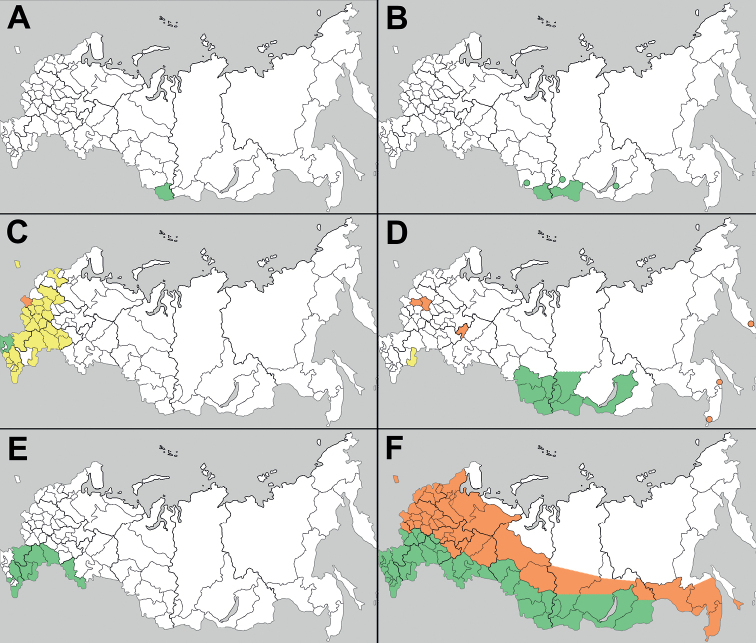
Schematic distribution areas of C_4_-species of *Atriplex* native to Russia **A***A.altaica***B***A.centralasiatica***C***A.rosea***D***A.sibirica***E***A.sphaeromorpha***F***A.tatarica*. Green – native distribution range, yellow – alien distribution not confirmed after 1930s, orange – alien distribution confirmed after 1930s.

Members of the section occur in steppes, semi-deserts and mountains of Central Asia (e.g. *A.altaica*, *A.centralasiatica*, *A.pamirica*, *A.sibirica*), in the Aralo-Caspian floristic region (*A.sphaeromorpha*), in the Mediterranean (*A.rosea*), and in North America (*A.argentea* Nutt.). Five species are native to Russia (*A.altaica*, *A.centralasiatica*, *A.sibirica*, *A.sphaeromorpha*, and *A.rosea*). The Central Asian species (*A.altaica*, *A.centralasiatica*, *A.sibirica*) are mostly confined to mountain steppes and screes in South Siberia, but *A.centralasiatica* and *A.sibirica* can be found also in saline and ruderal habitats. *Atriplexrosea* and *A.sphaeromorpha* are typical lowland species with similar morphology but different distribution patterns. The first species, with predominantly Mediterranean distribution, was considered native in the southern part of Eastern Europe (Medvedeva 1996), whereas we treat it as native only in Krasnodarsky Kray, where it occurs near the shore of the Black Sea and in ruderal places further inland (Sukhorukov 2006; Zernov 2006). *Atriplexsphaeromorpha* is mainly distributed in steppes and semi-deserts of Kazakhstan, with very scattered records in Orenburg and Saratov Oblast, as well as in the North Caucasus (Sukhorukov 1999, 2006). In these regions, *A.sphaeromorpha* could potentially require conservation action according to the IUCN guidelines (IUCN 2022) because of a high level of anthropogenic disturbance to Eurasian grasslands. The native and alien ranges of all Russian species of A.sect.Obione are displayed in Fig. 1A–E.

##### Taxonomic notes.

The synonymisation of Atriplexsect.Obione with A.sect.Sclerocalymma and A.subsect.Argenteae is undertaken here for the first time. *Atriplexpowellii* S.Watson, previously considered as a close relative of *A.argentea* (Standley 1916; Welsh 2001), occupies a distant phylogenetic position (Žerdoner Čalasan et al. 2022).

##### Nomenclatural notes.

The name A.sect.Obione has usually been credited to Meyer (1833), who accepted this subdivision in ‘Flora Altaica’. However, the first author who segregated this section within *Atriplex* was Reichenbach (1828). He accepted the name and provided an indirect reference to the basionym as “Obione. G.” The infrageneric taxa accepted by Reichenbach (1828) were explicitly ranked as sections, as evidenced by a footnote on page 184 in this work. Similarly, Hooker (1870) was the first to accept A.subgen.Obione, which was explicitly ranked by him. On the contrary, Ascherson (1864) developed complex infrageneric systems that included at least three ranks, with names at all of these ranks, and made no note of their ranking; such classifications are to be treated as unranked (Art. 37.3).

#### ﻿ Atriplex sect. Obionopsis

Taxon classificationPlantaeCaryophyllalesChenopodiaceae

(Lange) Dumort., Bull. Soc. Bot. Fr. 20: xiii (1873).

15BE0B63-5EB8-5F59-A4AB-79081470998F

≡ Atriplex [unranked] Obionopsis Lange, Haandb. Danske Fl., ed. 2, [7]: 635 (1859).Type species: Atriplexarenaria J.Woods 1849, non Nuttall 1818 (= Atriplexlaciniata L.).
= Atriplexsect.Psammophila Sukhor., Ann. Naturhist. Mus. Wien 108 B: 388 (2006), syn. nov. Type species: Atriplexdimorphostegia Kar. & Kir.

##### Description.

Annuals; inflorescences aphyllous or bracteate, rarely leafy in the lower and middle parts; glomerules loosely or densely arranged.

##### Species.

This section includes *A.dimorphostegia* Kar. & Kir., *A.fominii* Iljin, *A.kalafganica* Podlech, *A.laciniata* L., *A.lasiantha* Boiss., *A.olivieri* Moq., *A.ornata* Iljin, *A.paradoxa* Nikitina, *A.pratovii* Sukhor., *A.pungens* Trautv., *A.recurva* d’Urv., *A.schugnanica* Iljin, *A.tatarica* L., and *A.tornabenei* Tineo.

##### Native distribution.

Members of this section are distributed mostly in the Irano-Turanian floristic region, with extensions into the Mediterranean and Western Europe. *Atriplexparadoxa* is the only species native to Central Asia (Tian-Shan Mountains). Two species are present in Russia: *A.fominii* (not shown on the map), which is restricted to the western shore of the Caspian Sea in Dagestan and Azerbaijan (Iljin 1936; Sukhorukov 2006), and the widely distributed *A.tatarica* (Fig. 1F). In the steppe and desert zones of Russia, *A.tatarica* is considered native based on its presence in natural landscapes (mostly on saline soils).

##### Nomenclatural notes.

Lange (1859) used unranked infrageneric categories in his classification, whereas Du Mortier (1873) was apparently the first to explicitly rank *Obionopsis* as a section of *Atriplex*, but the nomenclatural significance of Du Mortier’s publication was overlooked.

The second edition of Lange’s ‘Haandbog i den Danske Flora’ was published in 7 parts during 1856–1859 (Stafleu and Cowan 1979). These parts were distributed separately by commercial publishers and are therefore effectively published, and the nomenclatural novelties in this work should be cited as published in its parts. The complete book bears no note on the parts and their dates, and no information on the book’s structure and dates is publicly available. According to contemporary announcements of book sales, the concluding part 7 of Lange’s book (with the treatment of Chenopodiaceae) was published in 1859 and consisted of 172 pages, which agrees with the printer’s signatures.

### ﻿Morphological notes on the members of Atriplexsect.Obione and A.sect.Obionopsis

Considering the latest phylogenetic results, many morphological characters like annual life form, ± significant fusion of the valves enclosing the female flowers, indurated lower part of the bract-like cover and heteromorphic seeds, which have been traditionally used in sectional delimitation (e.g., Aellen 1939; Sukhorukov 2006), should be considered as convergent and thus unreliable for the delimitation of Atriplexsect.Obione and A.sect.Obionopsis.

The fusion of the valves of the female flowers can vary considerably within a single species as observed, e.g., in *A.centralasiatica* (Grubov 1966). This species is characterised by dimorphic bract-like and smooth covers (with and without dorsal outgrowths), with the latter ones being less fused. The valve fusion was examined in other annual species of both sections (Sukhorukov 2006). In A.sect.Obione, the valves are connate to 1/3–1/2 of their length in most species, but to 2/3 or higher in *A.sibirica*. In A.sect.Obionopsis the valves are usually fused to 1/3–1/2 of their length, but in *A.dimorphostegia* and *A.ornata* they are free (except the stalks, which are always fused), herbaceous and not inflated.

Seeds are usually dimorphic, red and brown in most members of both sections (Sukhorukov 2006). In light of the recent phylogenetic results (Žerdoner Čalasan et al. 2022), the trimorphic (black, red and brown) seeds mentioned by Sukhorukov (2006) as a key character for A.sect.Psammophila (now included in A.sect.Obionopsis) as well as the stalked and herbaceous valves should be re-evaluated as rare synapomorphies among the C_4_-species of the genus.

Leafy inflorescences, which were considered characteristic of A.sect.Obione (Sukhorukov 2006), are also present in several species of A.sect.Obionopsis (*A.dimorphostegia*, *A.laciniata*, *A.ornata*, *A.pratovii*). We conclude that there are no specific morphological traits that can distinguish both sections.

### ﻿Notes on alien status of the species under consideration

**Figure 2.**
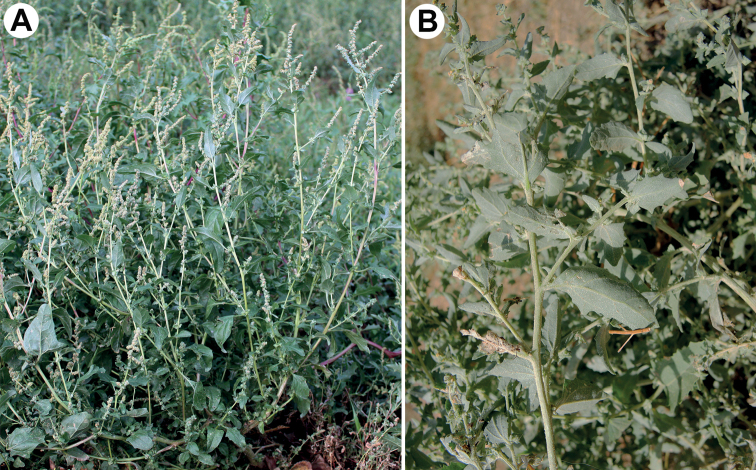
General view of the plants: **A***A.tatarica***B***A.rosea.* Photographs by A. Sukhorukov (**A** Russia, Tambov Oblast, Uvarovo, 20 September 2008) and M. Chambouleyron (**B** Morocco, Jerada, 23 August 2019).

From all the C_4_-species of *Atriplex* growing in Russia, only *A.tatarica* (Fig. 2A) seems to be able to spread continuously to the north reaching the forest zone (Fig. 1F). It is found in almost all regions of European Russia and Siberia up to 60°–65°N (e.g., Lomonosova 1992; Medvedeva 1996; Uotila 2011), with potential outposts in the Russian Far East (Ignatov 1988). In the forest zone, *A.tatarica* is usually found along the railway tracks on gravelly soil, where it can be classified as a typical “railway-wandering plant” (terminology after Kornaś et al. 1959). Nevertheless, sometimes it can also be encountered in towns as a ruderal plant (Sukhorukov 2014). In the northern part of Central Russia, it is a neophyte (Vinogradova et al. 2009) rather than a native species or an archeophyte as proposed by Uotila (2011). *Atriplextatarica* was included in the “Black Book [Invasive and naturalized alien plants] of the flora of Middle Russia” (Vinogradova et al. 2009), but its invasive status was apparently exaggerated, at least for the provinces situated in the forest zone, due to a limited number of suitable habitats in this area. In steppes of European Russia, *A.tatarica* seems to be native (but allochthonous) and can be found in a wide range of habitats including saline soils, same as in more southern regions of temperate Eurasia.

*Atriplexsibirica* is native to Central Asia and South Siberia (Sukhorukov 2006) where it grows in steppes, on loamy or stony soils, or as a ruderal plant. Surprisingly, this species is rarely found as an alien taxon in other regions despite the presence of suitable habitats, and almost all of the several recent findings outside of Siberia (Fig. 1D) are located along railway tracks. For example, in the Russian Far East it was found for the first time in 1973 in Kamchatka Kray (MHA0303450, as *A.rosea*) and correctly identified by Ignatov (1988), then collected once in Khabarovsky Kray in 1990 (MHA0303451). The first record of *A.sibirica* in Primorsky Kray is reported here: “Vladivostok town, Ugol’naya railway station, gravely substrate, 9 Aug 1988, *T. Nechaeva s.n*.” (MHA0303481, as *A.tatarica*). Among several sheets collected from this locality by T. Nechaeva, only one specimen belongs to *A.sibirica*, whereas all other specimens were correctly identified as *A.tatarica*. In European Russia, *A.sibirica* was known only from two provinces (Sukhorukov 2014; Fig. 1D in the present paper). Because of its scattered records in both Far East and European Russia, *A.sibirica* should be considered a casual alien without naturalization potential.

Contrary to *A.sibirica* and *A.tatarica*, the secondary range of *A.rosea* (Fig. 2B) in Eastern Europe has dramatically declined (Sukhorukov 2006). Almost all the recent claims about its wide distribution in the central and south parts of European Russia are erroneous, and since the 1930s there were no new records of *A.rosea* except the occurrences in Krasnodarsky Kray, Crimea (MW! MHA! see also Sukhorukov 2006) and Bryansk Oblast (MSK!), the territories with mild climatic conditions. The causes of its disappearance are still unknown, and further observations are needed to revise the distribution and ecological preferences of *A.rosea* in the countries bordering the Black Sea.

### ﻿A new record of an alien C_4_-species from Russia

Among the Russian specimens of *Atriplexprostrata* Boucher ex DC. (A.sect.Teutliopsis Dumort.: Sukhorukov 2006; Žerdoner Čalasan et al. 2022), which are deposited at MW, an unusual plant was found that readily differs from this species by its (sub)opposite leaves with crenate blades and by the leaf venation with remarkable chlorenchyma stripes that is peculiar for the atriplicoid Kranz anatomy. These features indicate that this plant is actually *A.flabellum*, a desert species from Central Asia, Iran and Afghanistan, which has never been reported for Russia (Lomonosova 1992; Sukhorukov 2006). This record is treated in detail here, separate from the other C_4_-species because of its novelty and casual non-native origin.

#### ﻿ Atriplex flabellum

Taxon classificationPlantaeCaryophyllalesChenopodiaceae

Bunge in Boiss., Fl. Orient. 4: 912 (1879).

F29764A8-B47D-5855-B726-4F818CD797AC

≡ Obioneflabellum (Bunge) Ulbr. in Engler & Prantl, Nat. Pflanzenfam., ed. 2, 16c: 506 (1934). Type: Iran. “Persia, in montosis salsis ad orientem urbis Meschhed, inter Faz et Tabatkuh prov. Khorassan”, *A. Bunge* (LE!, lectotype designated by Sukhorukov (2006: 408)).

##### Morphology.

For a detailed description, see Iljin (1936), Hedge (1997) and Sukhorukov (2006). The species is recognisable in all stages by its (sub)opposite crenate leaves with the Kranz-type anatomy, and flabellate and stalked bract-like covers of the female flowers with ventrally fused valves. The (sub)opposite leaves are very rarely found in the C_4_-clade of *Atriplex*, and the ventral valve fusion is present only in two Eurasian species of the genus, *A.flabellum* and *A.moneta*.

##### Specimen seen

**Figure 3.**
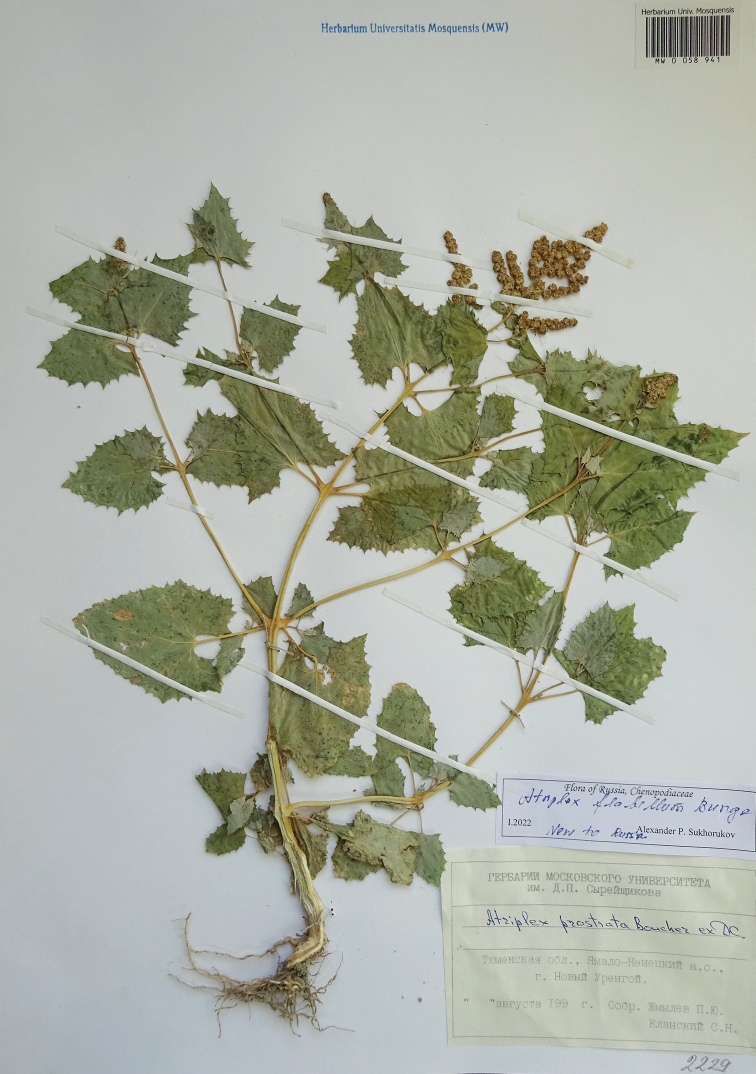
The voucher specimen of *Atriplexflabellum* recorded in Russia.

**(Fig. 3).** Russia, Tyumen Oblast, Yamalo-Nenetsky Autonomous District, Novy Urengoy Town, Aug 199X [year unknown], *P. Zhmylyov & S. Elansky s.n.* (MW0058941!). Image available at https://plant.depo.msu.ru/public/scan.jpg?pcode=MW0058941.

##### Habitat.

In Russia, the species occupies ruderal habitats. Within its native distribution range, it occurs in the desert zone on sandy and loamy soils in lowlands and foothills.

##### Introduction status.

Casual alien. *Atriplexflabellum* is a typical desert plant, and its populations cannot become established in the extreme north of the boreal zone. For this reason, we presume that this population is most likely extinct now.

##### Native distribution.

Afghanistan, Iran, Kazakhstan (south and south-east), Kyrgyzstan, Tajikistan, Turkmenistan, Uzbekistan.

##### Alien distribution.

**Figure 4.**
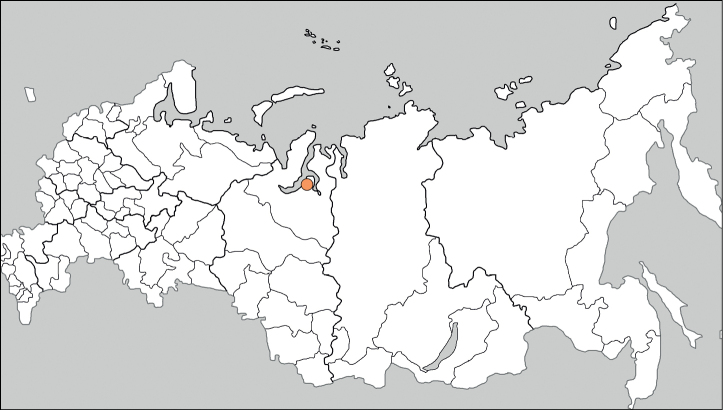
The location of a single alien record of *Atriplexflabellum* in Russia.

Russia (Tyumen Oblast) (Fig. 4).

##### Taxonomy.

The phylogenetic position of *A.flabellum* is distant to both A.sect.Obione and A.sect.Obionopsis; this species belongs to the basal grade within a large clade encompassing the majority of the Old World species of the genus (Žerdoner Čalasan et al. 2022). It was assigned to A.subgen.Pterotheca (Aellen) Sukhor. (lectotype species: *A.moneta* Bunge (Sukhorukov 2006)), whose monophyly has not been confirmed, and the sectional placement of *A.flabellum* has not been evaluated. A new classification of *Atriplex* is currently in preparation by Sukhorukov et al.

This Russian record of *Atriplexflabellum* is unexpected. Two more species of the C_4_-clade of *Atriplex*, *A.dimorphostegia* and *A.pungens* (both belonging to A.sect.Obionopsis), occur in West Kazakhstan at their western distribution limit (more detail in Sukhorukov 2006) and can potentially be found in adjacent regions of Russia (e.g., Astrakhan Oblast) as alien or sporadically distributed native plants.

### ﻿Key to the native and alien C_4_-species of *Atriplex* growing in Russia

**Table:** 

1	Leaves (sub)opposite, crenate; valves at fruiting flabellate, stalked, ventrally fused	***A.flabellum***
–	Leaves alternate, of different shape; valves marginally fused	**2**
2	Inflorescence leafy (almost) to the top; leaves rhombic, triangular or spatulate, entire to lobate	**3**
–	Inflorescence not leafy, sometimes one to several leaves present; leaves of different shape (linear, lanceolate or rhombic), entire to sinuate	**7**
3	Plants small (up to 20–30 cm), not forming a tumble-weed habit; bract-like cover dorsally without outgrowths	***A.altaica***
–	Plants forming a tumble-weed or spreading habit; bract-like cover usually with outgrowths	**4**
4	At least some valves of female flowers stalked, with thorn-like outgrowths located along the seed-containing part; plants native to Siberia, rarely found in other regions as aliens	**5**
–	Valves sessile, with one to several outgrowths located near their centre, rarely smooth; plants native to Europe	**6**
5	Valves monomorphic, all with thorn-like outgrowths	***A.sibirica***
–	Valves dimorphic, smooth and with thorn-like outgrowths on the same plant	***A.centralasiatica***
6	Inflorescence branches almost filiform; each cluster with 1–3 female flowers; steppe plants	***A.sphaeromorpha***
–	Inflorescence branches not filiform, stout; each cluster with 3–6 female flowers; ruderal or coastal habitats	***A.rosea***
7	Inflorescence bracteate; bract-like cover not inflated	***A.tatarica***
–	Inflorescence leafy in its lower and middle part; bract-like cover inflated	***A.fominii***

## ﻿Conclusions

A new, phylogeny-based classification of the C_4_-species of *Atriplex* occurring in Russia, places them into two large groups, which are morphologically similar but geographically rather distinct.

The phylogenetic circumscription of these groups shows that many characters that evolved in these lineages are highly convergent; thus it is impossible to find any clear morphological differences between these lineages. They can be characterised by different tendencies in certain diagnostic characters.

This classification is the first step towards a new phylogeny-based revision of the taxonomy of *Atriplex* worldwide. In addition to the gaps in the recent phylogenetic studies, for which some important species have not been sampled yet, a significant difficulty is presented by the vast corpus of old taxonomic literature, which has never been evaluated for the infrageneric nomenclature.

Many *Atriplex* species readily colonise disturbed habitats and spread widely to new territories next to or even far away from their native distribution areas. In addition to the first record of *A.flabellum*, further records of non-native species are expected in Russia, especially those with the ranges located close to the country, e.g. *A.dimorphostegia* and *A.pungens*.

## Supplementary Material

XML Treatment for Atriplex sect. Obione

XML Treatment for Atriplex sect. Obionopsis

XML Treatment for Atriplex flabellum

## References

[B1] Aellen P (1939) Die *Atriplex*-Arten des Orients. Botanische Jahrbücher für Systematik, Pflanzengeschichte und Pflanzengeographie 70(1): 1–66.

[B2] APG IV (2016) An update of the Angiosperm Phylogeny Group classification for the orders and families of flowering plants: APG IV. Botanical Journal of the Linnean Society 181(1): 1–20. 10.1111/boj.12385

[B3] Ascherson PFA (1864) Flora der Provinz Brandenburg, der Altmark, und des Herzogthums Magdeburg, Vol. 1, part 2. A.Hirschwald, Berlin, 321–1034.

[B4] Carolin RC Jacobs SWL Vesk M (1975) Leaf structure in Chenopodiaceae. Botanische Jahrbücher für Systematik, Pflanzengeschichte und Pflanzengeographie 95(2): 226–255.

[B5] Du Mortier B-C (1873) Note sur l’*Atriplexlaciniata* de Linné. Bulletin de la Société Botanique de France 20(sup1): xiii–xvi. 10.1080/00378941.1873.10839538

[B6] Fuentes-Bazan S Uotila P Borsch T (2012) A novel phylogeny-based generic classification for *Chenopodium* sensu lato, and a tribal rearrangement of Chenopodioideae (Chenopodiaceae). Willdenowia 42(1): 5–24. 10.3372/wi.42.42101

[B7] Grubov VI (1966) Plants of Central Asia, Vol. 2. Academy of Sciences of the USSR, Moscow & Leningrad. [In Russian]

[B8] Hedge IC (1997) *Atriplex*. In: Rechinger KH (Ed.) Flora Iranica, Vol.197. Akademische Druck- und Verlagsanstalt, Graz, 63–87.

[B9] Hooker JD (1870) The student’s flora of the British Islands. Macmillan & Co., London, 1–504. 10.5962/bhl.title.19896

[B10] Ignatov MS (1988) Chenopodiaceae. In: Kharkevich SS (Ed.) Sosudistye Rasteniya Sovetskogo Dal’nego Vostoka [Vascular plants of the Soviet Far East], Vol.3. Nauka, Leningrad, 15–37. [In Russian]

[B11] Iljin MM (1936) Chenopodiaceae. In: Shishkin BK (Ed.) Flora of the USSR, Vol.6. Academy of Sciences of the USSR, Moscow & Leningrad, 2–354. [In Russian]

[B12] IUCN (2022) Guidelines for Using the IUCN Red List Categories and Criteria. http://www.iucnredlist.org/documents/RedListGuidelines.pdf

[B13] Kadereit G Zacharias E Mavrodiev E Sukhorukov AP (2010) Molecular phylogeny of Atripliceae (Chenopodioideae, Chenopodiaceae): Implications for systematics, biogeography, flower and fruit evolution, and the origin of C_4_ photosynthesis. American Journal of Botany 97(10): 1664–1687. 10.3732/ajb.1000169 21616801

[B14] Kornaś J Leśniowska I Skrywanek A (1959) Obserwacja nad florą linii kolejowych i dworców towarowych w Krakowie. Fragmenta Floristica et Geobotanica Polonica 5(2): 199–216.

[B15] Lange JMC (1859) Haandbog i den Danske Flora, 2^nd^ edn., part 7. Reitzel, Kjøbenhavn, 593–764.

[B16] Lomonosova MN (1992) *Atriplex*. In: Krasnoborov IM Malyshev LI (Eds) Flora of Siberia, Vol.5. Science Publishers, Novosibirsk, 150–157. [In Russian]

[B17] Medvedeva NA (1996) *Atriplex*. In: Tzvelev NN (Ed.) Flora of Eastern Europe, Vol.9. World and Family-95, Saint-Petersburg, 44–54. [In Russian]

[B18] Meyer CA (1833) *Atriplex* L. In: von Ledebour CF Meyer CA von Bunge A (Eds) Flora Altaica, Vol.4. G.Reimer, Berlin, 304–318.

[B19] Moser H (1934) Untersuchungen über die Blattstruktur von *Atriplex*-Arten und ihre Beziehungen zur Systematik. Beihefte zum Botanischen Centralblatt 52B(2): 378–388.

[B20] Reichenbach HGL (1828) Übersicht des Gewächs-Reichs in seinen natürlichen Entwicklungsstufen. C. Cnobloch, Leipzig, 1–295. 10.5962/bhl.title.127510

[B21] Sage RF (2016) A portrait of the C_4_ photosynthetic family on the 50^th^ anniversary of its discovery: Species number, evolutionary lineages, and Hall of Fame. Journal of Experimental Botany 67(14): 4039–4056. 10.1093/jxb/erw156 27053721

[B22] Stafleu FA Cowan RS (1979) Taxonomic literature. A selective guide to botanical publications and collections with dates, commentaries and types, vol. 2. Bohn, Scheltema & Holkema, Utrecht & Antwerpen; dr. W. Junk b.v., Publishers, The Hague.

[B23] Standley PC (1916) Chenopodiaceae. In: Britton NL (Ed.) North American Flora, Vol.21(1). New York Botanical Garden, New York, 1–93.

[B24] Sukhorukov AP (1999) On the distribution of *Atriplexsphaeromorpha* Iljin (Chenopodiaceae). Bulletin of the Moscow Society of Naturalists. Biology 104(6): 58–59. [In Russian]

[B25] Sukhorukov AP (2006) Zur Systematik und Chorologie der in Russland und den benachbarten Staaten (in den Grenzen der ehemaligen USSR) vorkommenden *Atriplex*-Arten (Chenopodiaceae). Annalen des Naturhistorischen Museums in Wien 108 B: 307–420.

[B26] Sukhorukov AP (2014) The carpology of the Chenopodiaceae with reference to the phylogeny, systematics and diagnostics of its representatives. Grif & co., Tula, 1–400. [in Russian]

[B27] Turland NJ Wiersema JH Barrie FR Greuter W Hawksworth DL Herendeen PS Knapp S Kusber W-H Li D-Z Marhold K May TW McNeill J Monro AM Prado J Price MJ Smith GF [Eds] (2018) International Code of Nomenclature for algae, fungi, and plants (Shenzhen Code): Adopted by the Nineteenth International Botanical Congress, Shenzhen, China, July 2017. Regnum Vegetabile 159. Koeltz Botanical Books, Glashütten. 10.12705/Code.2018

[B28] Uotila P (2011) Chenopodiaceae (pro parte majore). Euro+Med Plantbase – the information resource for Euro-Mediterranean plant diversity. https://ww2.bgbm.org/EuroPlusMed/results.asp

[B29] Vinogradova YK Mayorov SL Khorun LV (2009) Chornaya kniga flory Sredney Rossii [Black book of the flora of the Middle Russia]. GEOS, Moscow, 1–494. [in Russian]

[B30] Welsh SL (2001) Nomenclatural proposals in *Atriplex* (Chenopodiaceae). Rhodora 102: 415–427.

[B31] Wilson PG (1984) Chenopodiaceae. In: George AS (Ed.) Flora of Australia, Vol.4. Australian Government Publishing Service, Canberra, 81–317.

[B32] Žerdoner Čalasan A Hammen S Sukhorukov AP McDonald JT Brignone NF Böhnert T Kadereit G (2022) From continental Asia into the world: Global historical biogeography of the saltbush genus *Atriplex* (Chenopodieae, Chenopodioideae, Amaranthaceae). Perspectives in Plant Ecology, Evolution and Systematics 54: e125660. 10.1016/j.ppees.2022.125660

[B33] Zernov AS (2006) Flora of the North-West Caucasus. KMK Scientific Press, Moscow, 664 pp. [In Russian]

